# Population structure and historical demography of South American sea lions provide insights into the catastrophic decline of a marine mammal population

**DOI:** 10.1098/rsos.160291

**Published:** 2016-07-27

**Authors:** J. I. Hoffman, G. J. Kowalski, A. Klimova, L. J. Eberhart-Phillips, I. J. Staniland, A. M. M. Baylis

**Affiliations:** 1 Department of Animal Behaviour, University of Bielefeld, Postfach 100131, 33501 Bielefeld, Germany; 2 Animal Ecology Group, Institute of Biochemistry and Biology, University of Potsdam, Maulbeerallee 1, 14469, Potsdam, Germany; 3 Centro de Investigaciones Biológicas del Noroeste Baja California Sur, La Paz, Mexico; 4 British Antarctic Survey, Natural Environment Research Council, High Cross, Madingley Road, Cambridge CB3 0ET, UK; 5 South Atlantic Environmental Research Institute, Stanley FIQQ1ZZ, Falkland Islands; 6 Falklands Conservation, Stanley FIQQ1ZZ, Falkland Islands; 7 Department of Biological Sciences, Macquarie University, Sydney, New South Wales 2109, Australia

**Keywords:** population structure, anthropogenic exploitation, historical demography, phylogeography, pinniped

## Abstract

Understanding the causes of population decline is crucial for conservation management. We therefore used genetic analysis both to provide baseline data on population structure and to evaluate hypotheses for the catastrophic decline of the South American sea lion (*Otaria flavescens*) at the Falkland Islands (Malvinas) in the South Atlantic. We genotyped 259 animals from 23 colonies across the Falklands at 281 bp of the mitochondrial hypervariable region and 22 microsatellites. A weak signature of population structure was detected, genetic diversity was moderately high in comparison with other pinniped species, and no evidence was found for the decline being associated with a strong demographic bottleneck. By combining our mitochondrial data with published sequences from Argentina, Brazil, Chile and Peru, we also uncovered strong maternally directed population structure across the geographical range of the species. In particular, very few shared haplotypes were found between the Falklands and South America, and this was reflected in correspondingly low migration rate estimates. These findings do not support the prominent hypothesis that the decline was caused by migration to Argentina, where large-scale commercial harvesting operations claimed over half a million animals. Thus, our study not only provides baseline data for conservation management but also reveals the potential for genetic studies to shed light upon long-standing questions pertaining to the history and fate of natural populations.

## 1. Introduction

Conservation genetics can contribute towards the management of threatened wildlife species in numerous ways [1,2]. For example, an important goal of many studies is to elucidate the pattern and strength of population structure, as this can provide insights into factors that affect gene flow as well as inform conservation practitioners as to where best to invest resources in order to safeguard genetic diversity [3]. A related goal is to quantify the extent to which a focal population is connected to others by gene flow, as this may have implications for the retention of genetic diversity and the movement of beneficial alleles between populations [4]. Many conservationists are also concerned about levels of genetic diversity, which are often taken as a proxy for the potential of a population to adapt to environmental change [5,6]. However, to fully understand contemporary levels of genetic diversity requires an appreciation of the historical demographic processes that shaped this diversity.

The Pinnipedia, a group of 33 extant species of marine mammal comprising the true seals, eared seals and the walrus, have been subjected to numerous genetic studies. This is partly because they provide interesting case studies for understanding the effects of historical exploitation on contemporary levels of genetic diversity and population persistence. In particular, many populations of eared seal were reduced to low enough densities to be considered extinct [7], yet some are recovering to pre-exploitation numbers and have retained high levels of genetic diversity [8–12], whereas others have failed to stage a recovery.

The South American sea lion (*Otaria flavescens*) can be found in breeding colonies along the coasts and offshore islands of South America from Peru to Uruguay, as well as in the Falkland Islands (Malvinas) in the South Atlantic. At the Falklands, this species experienced a major decline, with pup production falling from over 80 000 in the 1930s [13] to less than 6000 in 1965 [14]. Sea lion numbers continued to decline into the 1990s, but have since recovered a little over the past two decades, although a range-wide survey in 2014 reported a pup production of 4500, which is lower than in 1965 and only around 6% of the 1930s estimate [15].

A number of hypotheses have been proposed to explain the initial decline and subsequent lack of recovery of the South American sea lion population at the Falkland Islands. One possibility is that the population crash was caused by commercial sealing at the Falklands, although historical records suggest that around 60 000 animals were killed between 1928 and 1966, which is nowhere near enough to account for the population decline [15,16]. However, over half a million sea lions were killed over the same period in Argentina [17] and various modelling exercises have reached different conclusions as to whether the Argentinian population could have sustained the reported level of exploitation without receiving migrants from other localities [15,16]. This led Thompson *et al.* [16] to propose that the decline of the Falklands population could be explained by combined sealing operations in the Falkland Islands and Argentina.

Several authors have also questioned why the population failed to recover to its original numbers despite the cessation of commercial sealing in 1966. Various factors could potentially have played a role including predation, disease and competition with commercial fisheries [15,18], but data are largely lacking with which to evaluate their potential importance. Another possibility supported by a recent analysis of sea surface temperature changes over the past two centuries is that environmental change could have impacted population growth by altering patterns of food availability [15]. Yet another possibility is that the species could have experienced a genetic bottleneck, leading to the loss of genetic diversity and adaptive potential. Although it seems unlikely that anthropogenic exploitation would have been severe enough to result in an appreciable loss of diversity, the census estimates are imperfect and it is also plausible that a bottleneck could have occurred prior to human habitation of the Falklands.

South American sea lions have been the focus of a number of previous genetic studies, which have provided valuable insights into population structure and levels of genetic diversity [19–23]. However, these studies have tended to focus on specific areas within South America such as Patagonia and Uruguay, leading Artico *et al.* [19] to call for a range-wide genetic survey. Furthermore, only one study included the Falkland Islands [20] and the sample sizes involved were arguably too small (14 samples from the west and 5 samples from the east) either to be informative about population structure within the Falklands or to allow firm conclusions to be reached about connectivity to the South American mainland.

Here, we generated sequence data for 259 animals from 23 colonies across the Falklands at 281 bp of the mitochondrial hypervariable region and genotyped 22 microsatellites in order to elucidate baseline patterns of population genetic structure and to explore the historical demography of the population. By combining our mitochondrial data with previously published sequences from the South American mainland, we also documented range-wide patterns of maternally directed population structure, looked for evidence of shared haplotypes between the Falkland Islands and South America, and estimated migration rates in order to test support for the hypothesis that the population decline was caused by the hunting of animals that migrated to Argentina.

## 2. Material and methods

### 2.1. Tissue sample collection and DNA extraction

**Figure 1.**
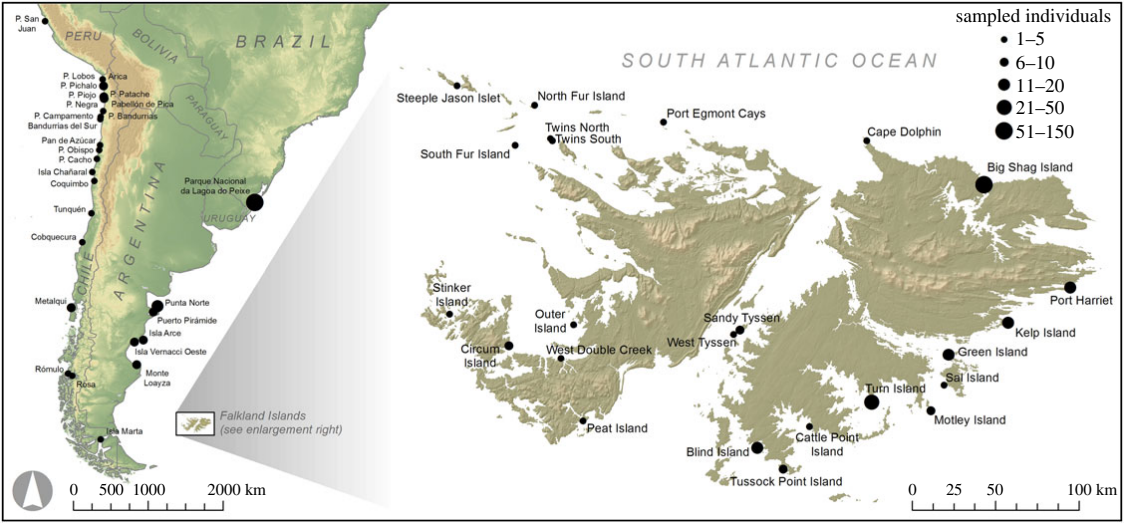
Map of the Falkland Islands showing the locations of South American sea lion breeding colonies from which genetic samples were obtained (see table 1 for details). Also shown is a map of South America, annotated with the sampling locations corresponding to 182 previously published mitochondrial control region sequences. Circle sizes are proportional to sample sizes in both panels.

Skin biopsy samples were collected from 277 live pups at 23 breeding colonies across the Falkland Islands (figure 1; electronic supplementary material, table S1). Pups were captured by hand and skin samples were taken adjacent to the distal phalange of the right hind flipper using a livestock ear notcher. The samples were stored individually in the preservative buffer 20% dimethyl sulfoxide (DMSO) saturated with salt and kept at –20°C. Total genomic DNA was extracted using a modified phenol–chloroform protocol [24].

### 2.2. Mitochondrial control region sequencing

A 365 bp region of the mitochondrial control region was PCR amplified using Thr/Pro (5′-TCCCTAAGACTCAAGGAAGAG-3′) and Cent (5′-GAGCGAGAAGAGGTACACTTT-3′) following Wynen *et al.* [25]. Each PCR was carried out in a 10 µl reaction volume containing 10 ng of template DNA, 0.25 µl Cent, 0.25 µl Thr/Pro, 0.1 µl Taq polymerase, 0.25 µl dNTPs, 7.15 µl of water and 1 µl of PCR buffer. The following PCR profile was used: one cycle of 15 min at 94°C; 30 cycles of 30 s at 94°C, 60 s at 60°C, 60 s at 72°C and a final cycle of 7 min at 72°C. Afterwards, 5 µl of PCR product was purified using Antarctic phosphatase and exonuclease I (New England Biolabs) following the manufacturer's recommended protocol. Samples were then sequenced using the Applied Biosystems BigDye® Terminator v. 3.1 Cycle Sequencing Kit and analysed on an ABI 3730xl capillary sequencer. All fragments were sequenced in both directions and consensus sequences were generated using ChromasPro v. 1.7.6 (Technelysium, Australia). The sequences were then aligned manually within BioEdit v. 7.1 [26]. Sites containing insertions/deletions or missing data were removed, including a highly variable region enriched for GA repeats that was previously described by Wynen *et al.* [25]. All of the sequences were then trimmed to the length of the shortest sequence, yielding 281 bp of contiguous sequence. All nucleotide positions differing from the consensus sequence were inspected to verify base calls were of high quality.

### 2.3. Microsatellite genotyping

After testing 48 microsatellite loci for PCR amplification in eight sea lion individuals, we selected 22 loci with polymorphic and unambiguous banding patterns for genotyping the full sample set (see table 1 for details). These were PCR amplified in five separate multiplexed reactions using a Type It Kit (Qiagen). We used a touchdown PCR profile as follows: one cycle of 5 min at 94°C; seven cycles of 30 s at 94°C, 45 s at 60–55°C (Δ*t *= −1°C) and 60 s at 68°C; 30 cycles of 30 s at 94°C, 45 s at 55°C and 60 s at 68°C and one final cycle of 30 min at 60°C. Fluorescently labelled PCR products were then resolved by electrophoresis on an ABI 3730xl capillary sequencer and allele sizes were scored using GeneMarker v. 1.95. To ensure high genotype quality, all traces were manually inspected and any incorrect calls were adjusted accordingly.

**Table 1.** Details of the 22 microsatellite loci employed in this study and their polymorphism characteristics in 259 Falkland Island sea lions. *N*_a_, number of alleles; *A*_e_, effective number of alleles; *H*_E_, expected heterozygosity; *H*_O_, observed heterozygosity. Individually significant Hardy–Weinberg equilibrium (HWE) *p*-values at *α* < 0.05 are highlighted in bold. None of these remained significant following table-wide FDR correction for multiple statistical tests.

locus	Mastermix	reference	*N*_a_	*A*_e_	*H*_E_	*H*_O_	null allele frequency	HWE *p*-value
Ag-7	3	[27]	6	2.40	0.59	0.56	0.00018	**0.046**
Ag-1	3	[27]	6	3.12	0.69	0.64	0.02267	0.196
Agaz-10	4	[28]	8	4.35	0.77	0.78	0.00003	0.411
Agaz-1	4	[28]	14	6.19	0.84	0.83	0.00669	0.233
Hg1.3	1	[29]	8	4.91	0.80	0.77	0.00827	0.547
Hg6.3	1	[30]	9	3.70	0.73	0.71	0.01773	0.178
OrrFCB8	4	[31]	6	3.75	0.73	0.71	0.02239	**0.049**
Hg8.10	1	[30]	21	9.14	0.89	0.86	0.01203	0.276
MHC26	5	[32]	8	5.92	0.83	0.83	0.00002	0.705
MHC4b	5	[32]	5	2.47	0.59	0.54	0.02925	0.288
ZcwE05	3	[31]	9	4.19	0.76	0.83	0.00000	0.447
OrrFCB7	2	[30]	7	3.16	0.68	0.62	0.04366	**0.003**
Pv9	1	[30]	8	4.1	0.76	0.76	0.00002	0.511
MHC11	4	[32]	4	1.99	0.50	0.49	0.02601	**0.005**
ZcCgDh1.8	4	[34]	4	1.71	0.41	0.41	0.00003	0.656
ZcwF07	4	[33]	10	3.90	0.74	0.76	0.00001	0.371
ZcCgDh4.7	3	[34]	11	3.95	0.75	0.75	0.00001	0.994
Agaz-6	2	[28]	4	1.63	0.39	0.39	0.00634	0.132
Ag-2	2	[27]	3	1.86	0.46	0.52	0.00001	0.075
OrrFCB2	2	[31]	11	2.17	0.54	0.56	0.01428	**0.032**
ZcCgDhB.14	2	[34]	5	2.41	0.59	0.59	0.00001	0.132
Ssl301	3	[35]	11	5.64	0.82	0.85	0.00009	0.055
overall			8.1	3.8	0.68	0.67	—	—

#### 2.3.1. Calculation of the genotyping error rate

In order to estimate the rate of microsatellite genotyping error, we independently repeat genotyped 20 samples. The resulting estimate was very low at 0.43% per locus or 0.22% per allele (four alleles incorrect out of 1856 comparisons).

### 2.4. Generation of summary statistics

The number of mitochondrial haplotypes, the number of polymorphic sites, haplotype diversity (*h*) and nucleotide diversity (*π*) were calculated using DnaSP v. 5.1 [36]. Haplotype frequencies were calculated using Arlequin v. 2.0 [37]. Genepop on the Web [38] was used to test each microsatellite locus for deviations from Hardy–Weinberg equilibrium. We set the dememorization number to 10 000, the number of batches to 1000 and the number of iterations per batch to 10 000. The resulting *p*-values were corrected table-wide for the false discovery rate (FDR) [39] using the program Q-value [40]. To calculate number of alleles, the effective number of alleles, observed and expected heterozygosities and to test for linkage disequilibrium, we used Fstat v. 2.9.3.2 [41] and GeneDive v. 2.0b23 [42]. Finally, we used FreeNA [43] to estimate null allele frequencies for each locus following the expectation maximization algorithm [44].

### 2.5. Population structure

Population structure within the Falkland Islands was assessed using hierarchical analyses of molecular variance (AMOVA) conducted within Arlequin. These analyses were carried out separately for the mitochondrial and microsatellite data at two different hierarchical levels: (i) comparing West and East Falkland and (ii) among the 23 breeding colonies. For the mitochondrial data, we used the measures *F*_st_ [45] and *Φ*_st_ [46], the former quantifying haplotype frequency differences [47] while the latter incorporates haplotype sequence similarity. For the microsatellite data, we used *F*_st_ [45] and *R*_st_ [48], the latter being a microsatellite-specific measure that takes account of the stepwise mutation process.

In order to test for population structure without knowledge of the sampling locations of individuals, we also conducted a Bayesian cluster analysis of the microsatellite dataset using Structure v. 2.3.3. [49]. This program uses a maximum-likelihood approach to cluster the genotypes into *K* populations. We ran five independent runs for *K* = 1 to 10 using 1 000 000 Markov chain Monte Carlo (MCMC) iterations after a burn-in of 100 000, the correlated allele frequencies model and assuming admixture. The most likely number of clusters was evaluated using the maximal average value of Ln *P*(*D*), a model-choice criterion that estimates the posterior probability of the data. As we have previously found that Structure results can be sensitive to the inclusion of individuals with substantial amounts of missing data, we restricted this analysis to 233 individuals genotyped at 20 or more loci.

### 2.6. Mitochondrial mismatch distribution

The distribution of the observed number of differences between each pair of haplotypes (the ‘mismatch distribution’) was calculated within Arlequin. This resembles a unimodal wave in samples drawn from recently expanded populations, whereas samples from static or bottlenecked populations tend to exhibit multimodal distributions [50,51]. Arlequin was also used to test for deviation of the observed dataset from a model of rapid population expansion assuming the same mean number of pairwise differences as the observed sample [51]. We also used DnaSP to test for deviations from neutrality with Tajima's *D* [52] and Fu's *F*_s_ [53]. Significant negative values of these statistics indicate an excess of low frequency polymorphisms, a pattern commonly attributed to recent population expansion.

### 2.7. Bottleneck tests

In order to investigate whether the Falkland Islands population underwent a genetic bottleneck, the microsatellite dataset was tested for heterozygosity excess [54,55] using Bottleneck v. 1.2.02 [56]. One criticism of this approach is that it can be sensitive to the mutational model assumed [57]. Although microsatellites mainly evolve according to the stepwise mutation model (SMM) in which a single repeat unit is gained or lost [58], multi-step mutations also occur [59,60]. We therefore specified a range of mutation models, from the strict SMM through two-phase models (TPMs) with varying proportions of multi-step mutations to the infinite alleles model (IAM) where every new mutation is novel. For our analysis, four TPM models were evaluated with 1%, 5%, 10% and 30% multi-step mutations respectively and a default variance of 30. For each of the mutational models, the heterozygosity of each locus expected under mutation-drift equilibrium given the observed number of alleles (*H*_eq_) was determined using 10 000 simulations and then compared against observed heterozygosity (*H*_e_). We then recorded the number of loci for which *H*_e_ was greater than *H*_eq_ and determined whether the overall set of deviations was statistically significant using standardized differences and Wilcoxon's signed rank tests. Bottlenecked populations are also expected to exhibit a characteristic ‘mode shift’ in the frequency distribution of alleles away from the L-shaped distribution expected under mutation-drift equilibrium [61]. Consequently, Bottleneck was also used to generate a qualitative descriptor of whether the observed allele frequencies at each locus deviate from such a distribution.

We also calculated Garza and Williamson's *M*-ratio for the microsatellite dataset using the program M_P_Val [62]. The significance of the resulting value was determined by comparison against *M*_c_, a critical value below which bottlenecks are inferred, using the program Critical_M [62]. This program allows the user to modify three parameters that approximate the mutation process in natural populations: the proportion of mutations that are larger than a single step (*p_g_*), the average size of non-single-step mutations (Δ*_g_*) and *θ* = 4*N*_e_*µ* (where *N*_e_ is the effective pre-bottleneck population size at equilibrium and *µ* is the mutation rate). We used the default settings of *p_g_* = 0.2 and Δ*_g_* = 3.5 [62] and varied *θ* between 1 and 1000, the latter corresponding to an effective pre-bottleneck population size of 500 000 assuming a commonly used estimate of the dinucleotide microsatellite mutation rate of 5 × 10^−4^ mutants per gamete per generation [63] as suggested by Garza & Williamson [62].

### 2.8. Approximate Bayesian computation

To further investigate the demographic history of the Falklands population, we used approximate Bayesian computation [64,65] as implemented in DIYABC v. 2.1.0 [66,67]. We tested support for four different demographic models describing different patterns of effective population size change over time. The first scenario that we evaluated (i) represented the null hypothesis of constant effective population size. The alternative scenarios invoked (ii) population expansion; (iii) population reduction and (iv) a bottleneck. Priors for the timing of events and the magnitude of changes of *N*_e_ were loosely based on knowledge of the demographic history of the species. For details of the models and priors used, please see electronic supplementary material, table S2. The microsatellite mutation rate was set between 5 × 10^−4^ and 5 × 10^−3^ substitutions per generation. The mitochondrial DNA mutation rate was bounded between 8.12 × 10^−7^ and 3.8 × 10^−6^ substitutions per site per generation [9,68]. We used four summary statistics for microsatellites (mean number of alleles, mean genic diversity, mean allele size variance and mean Garza and Williamson's *M*) and five summary statistics for the mitochondrial control region (number of haplotypes, number of segregating sites, Tajima's *D*, the number of private segregating sites and the mean number of the rarest nucleotide at the segregating site). These statistics were chosen on the basis of their sensitivity to demographic change. For each scenario, we simulated 1 × 10^6^ datasets separately for the microsatellite data, the mitochondrial data, and the combined microsatellite and mitochondrial data. After that, we used a polychotomous-weighted logistic regression on the 4 × 10^4^ simulated datasets closest to the observed dataset to determine the posterior probability for each scenario. In order to evaluate confidence in each scenario, we also calculated the posterior predictive error.

### 2.9. Genetic differentiation between the Falkland Islands and South America

In order to facilitate comparisons between the Falkland Islands and the South American mainland, we collated mitochondrial control region sequence data from published studies for which haplotype frequency data were available. Representative data were obtained for Argentina, Brazil, Chile and Peru (figure 1; electronic supplementary material, table S1). The sequences were downloaded from GenBank and then aligned to the Falkland Islands dataset within BioEdit. All the sequences were then adjusted to the length of the shortest sequence (254 bp). To provide a broad overview of the relationships among the mitochondrial haplotypes, we then constructed a median joining network using Network v. 4.516 [69]. This program calculates all possible minimum spanning trees for the dataset and then combines these into a single minimum spanning network using an algorithm analogous to that proposed by Excoffier & Smouse [70]. Inferred intermediate haplotypes are then added to the network in order to minimize its overall length. Finally, we used the mitochondrial data to conduct a formal assessment of the strength of population structure among the Falkland Islands, Argentina, Brazil, Peru and Chile. For the Falkland Islands, individuals from all 23 colonies were included and treated as one population. Pairwise *F*_st_ and *Φ*_st_ values were calculated and their significance determined using 10 000 permutations of the dataset.

### 2.10. Estimation of migration rates

Finally, we used Migrate v. 3.6.4 [71,72] to estimate migration rates and directions based on the mitochondrial data. Migrate uses Bayesian inference to estimate the posterior probability densities of migration rates and effective population sizes. For this analysis, we treated samples from Argentina, Brazil and Chile as distinct populations but excluded Peru due to the fact that only five sequences were available. After several exploratory runs, the final simulation was performed with one long chain and 100 replicates, where 125 000 000 steps were sampled, 50 000 were discarded as ‘burn-in’ and 50 000 steps were recorded. Prior values were bound between 0.0001 and 20 for Θ and between 0.0001 and 500 for *M* (mutation scaled migration rate). We used the recommended heating scheme (1.00, 1.50, 3.00, 1 000 000) and estimated the number of migrants per generation by multiplying the estimated mutation-scaled migration rate by the Θ value.

## 3. Results

Out of a total of 277 samples collected, we successfully sequenced a 281 bp region of the mitochondrial control region and genotyped 22 microsatellites in 259 sea lions from 23 different breeding colonies around the Falkland Islands (figure 1; electronic supplementary material, table S1). The mitochondrial control region contained 25 variable sites, all of which were parsimony informative, and a total of 22 haplotypes were identified, with nucleotide diversity being 0.015 and haplotype diversity being 0.864. The microsatellite loci carried on average 8.2 alleles, none deviated significantly from Hardy–Weinberg equilibrium after correction for multiple testing, and there was no evidence for the presence of null alleles (table 1).

### 3.1. Population structure within the Falkland Islands

AMOVA was used to determine the proportion of genetic variation attributable to each level of population substructure, separately for both mitochondrial DNA and microsatellites (table 2). Contrasting patterns were obtained for the two markers and these were somewhat dependent on the measure of genetic differentiation used. We did not find that a significant proportion of the variance in the mitochondrial data was partitioned at the uppermost hierarchical level, indicating a lack of differentiation between West and East Falkland. By contrast, among-colony variance components were highly significant using *F*_st_ and approached significance using *Φ*_st_. For microsatellites, significant differences were found using *F*_st_ between West and East Falkland as well as among the 23 colonies. However, none of the variance components were significant using *R*_st_ suggesting that the nuclear signal of population structure is rather weak.

**Table 2.** Results of analyses of molecular variance (AMOVA) based on (*a*,*b*) the mitochondrial control region and (*c*,*d*) 22 microsatellites, with the dataset being partitioned into West and East Falkland and 23 breeding colonies respectively. Significant *p*-values at *α* < 0.05 are highlighted in bold.

partition	source of variation	sum of squares	variance	% of total variance	*F*	*p*-value
(*a*) mitochondrial DNA (using *F*_st_)						
2 islands	among islands	0.56	−0.001	−0.34	−0.003	0.25
	among colonies within islands	12.49	0.02	4.63	0.05	**<0**.**001**
	within colonies	98.39	0.42	95.71	0.05	**<0**.**001**
23 colonies	among colonies	13.04	0.02	4.53	0.05	**<0**.**001**
	within colonies	98.39	0.42	95.47	—	—
(*b*) mitochondrial DNA (using *Φ*_st_)						
2 islands	among islands	3.31	0.009	0.46	0.01	0.09
	among colonies within islands	54.95	0.10	5.07	0.05	0.08
	within colonies	419.69	1.78	94.47	0.06	**0**.**02**
23 colonies	among colonies	58.25	0.10	5.21	0.05	**0**.**02**
	within colonies	419.69	1.78	94.79	—	—
(*c*) microsatellites (using *F*_st_)						
2 islands	among islands	12.21	0.03	0.44	0.004	**0**.**001**
	among colonies within islands	176.72	0.09	1.19	0.01	**<0**.**001**
	among individuals within colonies	1630.65	−0.15	−2.15	−0.02	0.98
	within individuals	1869.50	7.22	100.51	−0.01	0.89
23 colonies	among colonies	188.94	0.09	1.32	0.01	1.00
	among individuals within colonies	1630.65	−0.15	−2.16	−0.02	0.98
	within individuals	1869.50	7.22	100.84	−0.01	0.89
(*d*) microsatellites (using *R*_st_)						
2 islands	among islands	660.31	−0.00	−0.00	−0.00	0.12
	among colonies within islands	13 115.92	7.38	1.46	0.01	0.11
	among individuals within colonies	116 784.92	−3.67	−0.72	−0.01	0.55
	within individuals	130 066.00	502.19	99.26	0.01	0.45
23 colonies	among colonies	13 776.24	7.39	1.46	0.02	0.98
	among individuals within colonies	116 784.92	−3.67	−0.72	−0.72	0.55
	within individuals	130 066.00	502.19	99.26	0.01	0.45

Arguably, the most powerful tests of population structure need not rely on knowledge of the sampling locations of individuals. We therefore carried out a Bayesian clustering analysis of the microsatellite dataset using Structure [49]. Five runs were conducted for each possible number of clusters (*K*) from 1 to 10. The highest average log-likelihood value was associated with *K* = 1 (electronic supplementary material, figure S1) indicating that Structure could not detect any population structure within the Falkland Islands.

### 3.2. Historical demography of the Falklands population

**Figure 2.**
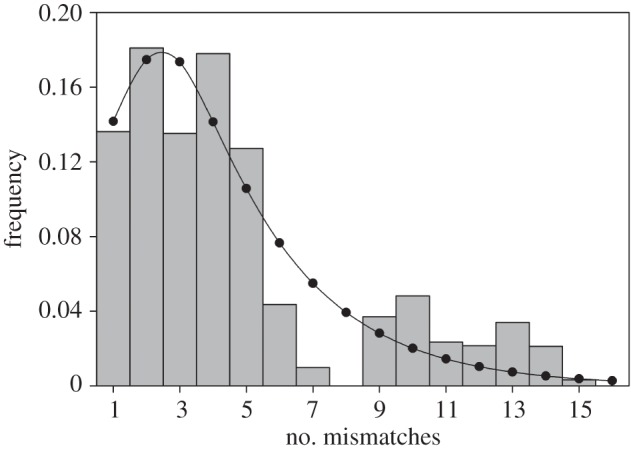
Histogram showing the observed distribution of pairwise differences among mitochondrial haplotypes obtained from the Falkland Islands. For comparison, the line with black points represents the expected distribution under a model of sudden population expansion.

To provide insights into the recent demographic history of South American sea lions at the Falkland Islands, we generated a mismatch distribution from the mitochondrial data (figure 2) and tested for deviation from a model of rapid population expansion. The sum of squared deviations between the observed and expected distribution and Harpending's raggedness index were both low and statistically insignificant (SSD = 0.009, *p* = 0.42; raggedness index = 0.019, *p* = 0.77) meaning that we could not rule out a model of population expansion. On the other hand, however, we also could not reject the null hypothesis of neutrality because Tajima's *D* and Fu's *F*_s_ values were negative but not statistically significant (*D* = −0.092, *p* = 0.48 and *F*_s_ = −0.084, *p* = 0.56 respectively).

To test the hypothesis that the population experienced a genetic bottleneck, we analysed the microsatellite data using the heterozygosity excess approach of Luikart & Cornuet [55]. The results varied with the mutational model on which the predicted relationship between heterozygosity and allele number was based (table 3). With models such as the SMM and TPMs with 5% or fewer multi-step mutations [59], no significant excess of heterozygosity was detected. Significance was only reached with the less conservative TPM with 30% multi-step mutations. A mode shift in the allele frequency distribution was not found.

**Table 3.** The number of loci with heterozygosity excess and test probabilities obtained using a range of mutational models within the program Bottleneck [56]. Significant *p*-values at *α* < 0.05 are highlighted in bold.

mutational model	no. loci with heterozygosity excess	sign test *p-*value	standardized differences test *p-*value	Wilcoxon's test *p-*value	Wilcoxon's test *p-*value for heterozygosity excess (one tailed)
IAM	21	**<0**.**0001**	**<0**.**0001**	**<0**.**0001**	**<0**.**0001**
TPM70	17	0.0550	0.1033	**0**.**0029**	**0**.**0014**
TPM90	11	0.2635	0.1166	0.6327	0.6949
TPM95	8	**0**.**0270**	**0**.**0044**	0.0501	0.9769
TPM99	5	**0**.**0006**	**<0**.**0001**	**0**.**0022**	0.9990
SMM	4	**0**.**0001**	**<0**.**0001**	**0**.**0004**	0.9999

For populations with large pre-bottleneck sizes, the ratio of the number of alleles to the allelic size range has been suggested to be more informative about bottleneck history than heterozygosity excess [73]. Consequently, we also calculated the *M*-ratio of Garza & Williamson [62]. The resulting value of 0.88 was above the 0.7 threshold proposed by Garza & Williamson [62] as well as the critical *M* values obtained through simulation (0.63–0.81), implying a lack of support for a bottleneck.

Finally, we analysed the mitochondrial and microsatellite data within an approximate Bayesian computation (ABC) framework to evaluate statistical support for the following four demographic scenarios: (i) constant population size; (ii) population expansion; (iii) population reduction and (iv) a population bottleneck (see electronic supplementary material, table S2 for further details). The best supported scenarios were population expansion for the mitochondrial dataset, stable population size for the microsatellite dataset and a bottleneck for the combined mitochondrial and microsatellite dataset (table 4). However, the posterior probabilities associated with the best supported scenarios were low (0.49, 0.39 and 0.36 respectively) and the corresponding posterior predictive error estimates were high (0.55, 0.58 and 0.35 respectively) indicating that this analysis is not very informative about the demographic history of the population.

**Table 4.** Posterior probability estimates for each competing scenario in the approximate Bayesian computation analysis based on three datasets. Posterior predictive errors are also shown for each analysis.

	posterior probability		
scenario	mitochondrial DNA	microsatellites	combined mitochondrial DNA and microsatelites
constant population size	0.34	0.39	0.28
population expansion	0.49	0.05	0.29
population reduction	0.16	0.28	0.06
bottleneck	0.01	0.26	0.36
posterior predictive error	0.55	0.58	0.35

### 3.3. Relationship to the South American mainland

**Figure 3.**
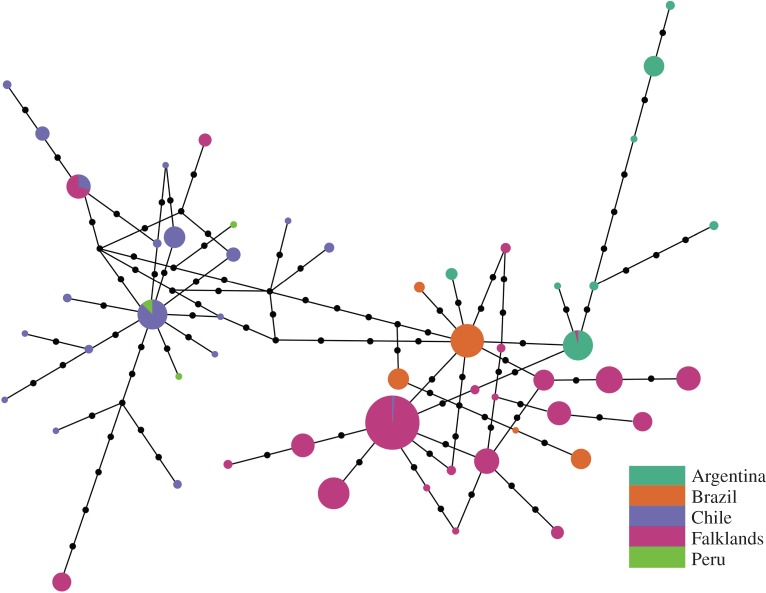
Median joining network showing the phylogenetic relationships among 53 mitochondrial haplotypes obtained from the Falkland Islands, Argentina, Brazil, Chile and Peru. Each line joining two circles corresponds to a single nucleotide substitution, with coloured circles representing observed haplotypes and black circles representing hypothetical haplotypes that were not observed in the sample. Circle size is proportional to the relative frequency of each of the observed haplotypes.

We analysed the relationship between sea lion colonies from the Falkland Islands and the South American mainland by comparing our mitochondrial data with 182 previously published mitochondrial sequences from Argentina, Brazil, Chile and Peru (figure 1; electronic supplementary material, table S1). For visualization, we generated a median joining network (figure 3). This revealed very strong maternally directed population structure across the geographical range of the species, which is reflected by large and highly significant pairwise *F*_st_ and *Φ*_st_ values (electronic supplementary material, table S3). From this figure it can also be seen that the Atlantic coast (Argentina and Brazil) and the Pacific coast (Chile and Peru) of South America are deeply divergent, with the Falkland Island haplotypes being more closely related to the former. In addition, two haplotypes were found to be shared between the Falkland Islands and Chile, and one between the Falkland Islands and Argentina, indicating a limited degree of maternally directed gene flow between the Falklands and the South American mainland.

### 3.4. Migration rates and directions

**Figure 4.**
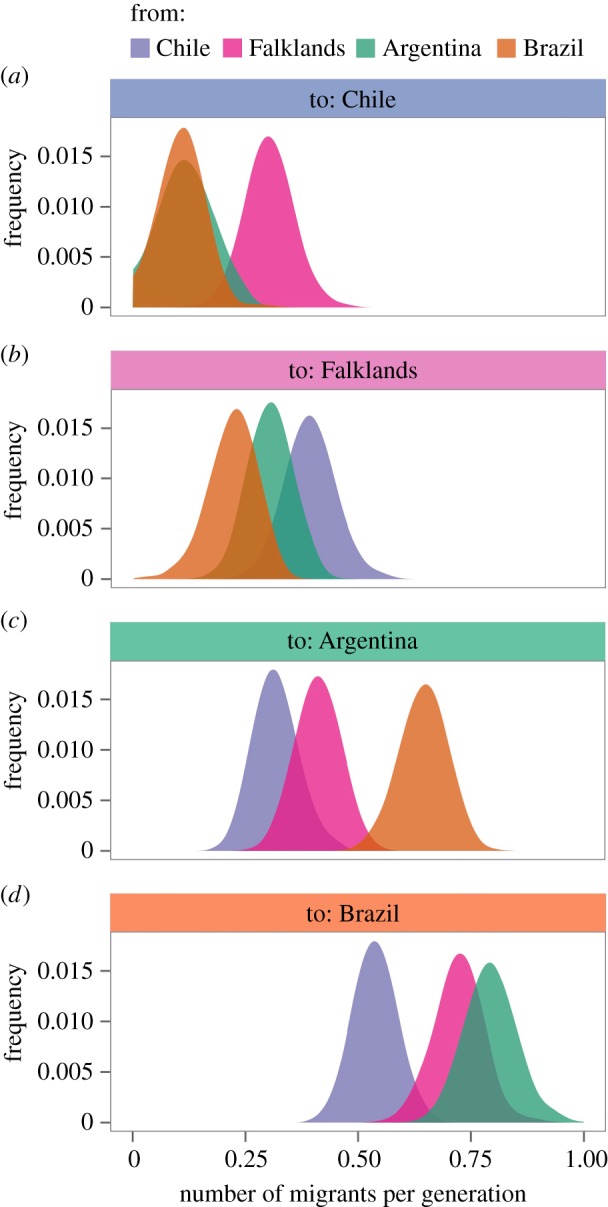
Migration rate estimates calculated from the mitochondrial control region using Migrate [71,72]. Depicted are distributions of migration rate estimates from Chile (blue), the Falklands (pink), Argentina (green) and Brazil (orange) towards (*a*) Chile; (*b*) the Falklands; (*c*) Argentina and (*d*) Brazil.

Finally, we used the program Migrate [71] to estimate rates and directions of gene flow from the mitochondrial data. Peru was excluded from this analysis due to the sample size being only five individuals. The resulting migration rate estimates were low, with all of the pairwise regional comparisons yielding estimates of less than one migrant per generation (figure 4; table 5). A tendency was also observed for Chile and the Falklands to receive fewer migrants from the other localities (figure 4*a* and *b* respectively) than Argentina and Brazil (figure 4*c* and *d* respectively). The two highest migration rate estimates were from Argentina to Brazil (green distribution in figure 4*d*) and from the Falklands to Brazil (pink distribution in figure 4*d*) possibly reflecting a combination of geographical proximity and prevailing currents (see Discussion).

**Table 5.** Modal estimates of gene flow among sea lion populations from the Falklands, Argentina, Brazil and Chile, calculated from the mitochondrial control region using Migrate [71,72]. Results are given as the number of migrants per generation from each of the areas on the left (row headings) into the areas on the right (column headings). 95% Confidence intervals are given in parentheses.

region	Falklands	Argentina	Brazil	Chile
Falklands	*	0.41 (0.31–0.51)	0.72 (0.59–0.83)	0.29 (0.19–0.42)
Argentina	0.31 (0.20–0.41)	*	0.79 (0.67–0.91)	0.11 (0.00–0.23)
Brazil	0.23 (0.11–0.33)	0.65 (0.53–0.75)	*	0.11 (0.01–0.20)
Chile	0.39 (0.27–0.51)	0.31 (0.21–0.42)	0.54 (0.44–0.64)	*

## 4. Discussion

We conducted a genetic analysis of South American sea lion populations around the Falkland Islands and combined our data with previously published mitochondrial sequences to allow a comparison to be made between the Falklands and the South American mainland. Within the Falkland Islands, we found moderately high levels of genetic diversity at both types of marker and no evidence for a recent genetic bottleneck. Population structure was also rather weak in comparison to the strong maternally directed population structure observed across the broader species range. Finally, migration rate estimates were very low in all pairwise comparisons involving Argentina, Brazil, Chile and the Falklands, which is at odds with the hypothesis that the decline was caused by historical exploitation in Argentina.

### 4.1. Population structure within the Falkland Islands

Tests for population structure within the Falkland Islands uncovered somewhat mixed results depending on the marker and genetic distance measure used. For this reason, and for consistency with previous studies of this species [20,21,23], our preferred approach was to partition the total genetic variance using AMOVA. For the mitochondrial DNA, we found that the uppermost hierarchical level of population structure was not statistically significant, implying a lack of differentiation between West and East Falkland. However, around 5% of the genetic variance could be attributed to among-colony differences, indicating the presence of shallow population structure. This variance component was highly significant using *F*_st_ but failed to reach significance using *Φ*_st_, suggesting that the overall result may be driven by haplotype frequency differences.

A similarly weak and rather inconsistent pattern was also obtained for the microsatellite data, despite our having used 22 loci. For this class of marker, a significant proportion of the genetic variance was attributable to differences between West and East Falkland as well as among colonies, but only using *F*_st_ and not *R*_st_. Similarly, Bayesian cluster analysis of the microsatellite dataset failed to uncover any evidence for population structure within the Falklands. Taken together, these results are suggestive of the presence of very weak population structure within the Falkland Islands, as a simulation study found that Structure often struggles to find sub-populations when *F*_st_ is below around 0.02 [74].

These findings are in line with previous studies of this species on the South American mainland. For instance, Túnez *et al.* [21] found weak among-colony differences in the mitochondrial control region in north-central Patagonia, whereas Crespo *et al.* [23] found no mitochondrial differences among populations sampled between Southern Brazil and Tierra del Fuego. In the only study of this species to have used both mitochondrial and nuclear markers, Feijoo *et al.* [20] found significant differences among colonies at the former but not the latter. This was interpreted as reflecting sex-biased dispersal, a pattern that is common among pinnipeds as males are often the dispersing sex [75–77]. This would be consistent with a tagging study of South American sea lions that documented long-distance movements of reproductively active adult males between Patagonia and Uruguay [78].

Our results are somewhat less straightforward to interpret, as genetic differences appear to be present among colonies but whether or not these achieve statistical significance depends on the genetic distance measure used. Moreover, taking our results at face value one might conclude that, although genetic differences among colonies are found at both class of marker, a difference between West and East Falkland is only apparent in the nuclear and not the mitochondrial genome. Such a pattern might be genuine, although this would be contrary to expectations based on strong female philopatry and male dispersal. Alternatively, it could be possible that different markers vary in their power to detect population structure [79]. To distinguish between these explanations as well as to resolve population structure more clearly, it would be desirable to increase both sample sizes and genetic resolution.

Regardless of the exact pattern of population structure, South American sea lions at the Falkland Islands show much weaker structuring than is evident over similar spatial scales in several other related pinniped species, including the Galápagos fur seal, *Arctocephalus galapagoensis* [77], the Galápagos sea lion, *Zalophus wollebaeki* [80] and the Australian sea lion, *Neophoca cinerea* [81]. This could have positive implications for conservation as there appears to be adequate gene flow between sea lion colonies within the Falklands both to counteract inbreeding and to maintain adaptive potential. The level of structure we observe is also insufficient to recommend the delimitation of evolutionary significant units (ESUs), which are often used to objectively define units below the level of the species that should be prioritized for conservation [82–84]. This is because ESUs should typically be reciprocally monophyletic for mitochondrial haplotypes and significantly divergent at nuclear loci [83]. However, a case could potentially be made for defining ESUs over a broader geographical scale due to the deep mitochondrial sequence divergence found among the Falkland Islands, Brazil, Argentina, Chile and Peru (see below).

### 4.2. Testing hypotheses for population decline

The first hypothesis we tested was that a historical bottleneck could have been involved in the failure of the sea lion population to recover from commercial exploitation. Although census data suggest that human exploitation may not have been severe enough to have depleted genetic diversity, these data are imperfect and effective population sizes are typically at least an order of magnitude smaller than census sizes, particularly in polygynous species such as sea lions where only a fraction of adults contribute towards successive generations [85]. Moreover, pinniped numbers are known to be highly responsive to changes in food or habitat availability [86–89], meaning that a bottleneck in the more distant past cannot be ruled out.

To test for a bottleneck, we analysed genetic data from the Falkland Islands using three complementary approaches. Although the results are perhaps not as clear cut as one might hope, a number of lines of evidence suggest that sea lions at the Falklands probably did not experience an appreciable loss of genetic diversity. First, we detected moderately high levels of genetic diversity at both mitochondrial DNA and microsatellites in comparison with values reported for 18 different pinniped species (see table 7 in [77]). Second, Bottleneck only found support for a bottleneck with the IAM and TPM with 30% multi-step mutations, while the more conservative TPMs with 5% or fewer multi-step mutations did not detect a significant excess of heterozygosity. Although this is difficult to interpret because the true proportion of multi-step mutations is unknown, a clear contrast can be drawn with the Antarctic fur seal (*Arctocephalus gazella*), which experienced a severe bottleneck that is reflected in a significant excess of heterozygosity with the TPM with 5% multi-step mutations [90]. Third, the empirical value of the *M*-ratio was not consistent with a bottleneck, and fourth, ABC analysis also failed to find convincing support for a bottleneck scenario. In contrast again with the Antarctic fur seal study, where ABC analysis conclusively favoured a bottleneck, we found that none of the four scenarios were robustly supported, either by the mitochondrial or microsatellite datasets on their own or when the two datasets were combined. One explanation for this stems from a recent analysis of the numbers and sexes of sea lions hunted in the Falklands from 1928 to 1966 [15]. Although over 60 000 animals were taken, the majority of these are thought to have been adult males, suggesting that any effects on breeding females may have been relatively minor.

A prominent hypothesis for the collapse of the sea lion population at the Falkland Islands is that large-scale commercial harvesting operations in Argentina could have been responsible [15,16,18,91]. However, for hunting in Argentina to have accounted for the 95% decline in pup production at the Falkland Islands, from approximately 80 000 in the mid-1930s to around 4500 today, a large fraction of the reproductively active female population of the Falklands would have had to have migrated to Argentina. Baylis *et al.* [15] argued that this is unlikely because females of this species lactate for around 11 months, leaving very little time for them to undertake long migrations. However, as the migration of subadults cannot be ruled out, we approached this hypothesis from a genetic perspective.

To explore patterns of genetic connectivity across the geographical range of the species, we analysed mitochondrial data from the Falklands, Argentina, Brazil, Chile and Peru. Only four shared haplotypes were found among these five regions, indicating very strong mitochondrial structuring over a continental scale. While this is consistent with a previous study of this species that compared Brazil with Peru [19], ours is the first study to document range-wide mitochondrial relationships, thereby revealing a broad tendency for restricted long-distance maternally directed dispersal. Such a pattern is broadly in line with previous large-scale studies of other pinniped species including the sympatric South American fur seal, *Arctocephalus australis* [92].

We also found limited evidence of haplotype sharing between the Falkland Islands and the South American mainland, with only two haplotypes being common to the Falklands and Chile, and one being shared by the Falklands and Argentina. By implication, maternally directed gene flow appears to be restricted between the Falklands and South America. This clearly goes against the notion that migration to Argentina could have been responsible for the decline of the Falklands population, although our genetic data would be unable to detect migration if the majority of migrants had been culled prior to breeding. This, however, seems unlikely for two main reasons. First, if female migration was a previously unappreciated aspect of this species biology, one would expect this to be reflected in widespread haplotype sharing due to the migration that would have taken place prior to commercial harvesting in Argentina. Second, the sea lion population in Argentina was estimated to have been almost twice the size of the Falklands population in the 1930s [93]. It is difficult to reconcile why sea lions from the Falklands would migrate to compete with a larger population of sea lions, particularly when considering that the Falkland Islands are within closer proximity to the Patagonian Shelf slope, a region of enhanced biological activity and productivity [94].

Consistent with the rarity of shared haplotypes, analysis of the global mitochondrial dataset within Migrate yielded very low migration rate estimates for all of the pairwise regional comparisons, lending further support to the notion of restricted long-distance female dispersal. We also found some evidence to suggest that migration rates towards Chile and the Falkland Islands may be lower on average than migration rates towards Argentina and Brazil, although some degree of caution is warranted due to the fact that all of the estimates are very low. Interestingly, the two highest estimates were from Argentina to Brazil and from the Falklands to Brazil. One possible interpretation of this could be that the prevailing northwards flow of the Malvinas current [95] may facilitate migration towards Brazil. It could be worthwhile investigating this further through biologging studies or targeted genetic studies.

Finally, because we do not have any microsatellite data from the South American mainland we cannot exclude the possibility of male-mediated gene flow from the Falkland Islands towards Argentina. However, it is unlikely that the harvesting of adult males would significantly influence population growth as pinniped population dynamics are typically most sensitive to the survival of adult and subadult females [89,96]. Moreover, although movements of reproductively active adult males have been documented within South America, Baylis *et al.* [15] found no evidence of males tagged at the Falklands migrating to Argentina [15]. Further biologging and genetic studies could shed light on this topic, although the latter would require tissue samples to be collected from representative locations around South America.

In conclusion, our study failed to find evidence of a strong bottleneck, consistent with the conclusion of Baylis *et al.* [15] based on historical records that anthropogenic impacts on the Falkland Island sea lion population may not have been as severe as previously thought. Our results are also at odds with the hypothesis that the decline could have resulted from females migrating to Argentina, where large-scale commercial harvesting operations were responsible for the deaths of over half a million sea lions. It therefore seems likely that local factors at the Falkland Islands, such as increased fisheries competition, disease, predation and/or environmental change may have played a role. Of these, Baylis *et al.* [15] argued that commercial fisheries are unlikely to have been an important driver of the decline because intensive near shore trawl fisheries were not developed until the 1980s. They instead showed that sea surface temperatures at the Falklands increased significantly during the period of steepest decline, suggesting a potential role of bottom-up trophic forcing on the sea lion population. Our results are consistent with this hypothesis in as far as commercial harvesting is unlikely to have caused the decline. Thus, to better understand the decline, it would seem appropriate for future studies to explore other potentially contributing factors, such as the impact of environmental variation on sea lion populations.

## Supplementary Material

Electronic supplementary material, figure S1. Results of the Structure [38] analysis showing average log-likelihood value values based on five replicates for each value of K, the hypothesized number of clusters in the data. Electronic supplementary material, table S1. Numbers of sea lion tissue samples collected and successfully sequenced for the mitochondrial control region and genotyped at 22 microsatellite loci. The sampling locations are shown in figure 1. For analyses within the Falkland islands, we only used samples that genotyped successfully at both the mitochondrial control region and at up to 22 microsatellites. Also shown are the number of previously published O. flavescens mitochondial control region sequences obtained from the literature corresponding to locations on the mainland of South America shown in figure 1. Electronic supplementary material, table S2. Prior distributions for demographic parameters in the approximate Bayesian computation analysis. N1, current effective population size; N2, effective population size before expansion; T1, time from expansion / reduction (generations ago); N3, effective population size before reduction; N4, effective population size during bottleneck; Npb, pre-bottleneck effective population size; T3, time for the end of the bottleneck; T3b, time for the start of the bottleneck. Electronic supplementary material, table S3. Estimates of genetic differentiation between the Falkland Islands and the South American mainland based on the mitochondrial control region. Pairwise Fst and Fst values are given above and below the diagonal respectively. FDR corrected P-values * P > 0.05; ** P >0.01; *** P > 0.001.

## Data accessibility

Mitochondrial DNA sequences are available from GenBank (accession nos. KU729822–KU729843). Microsatellite genotypes are available via Dryad at http://dx.doi.org/10.5061/dryad.d826h.
