# Supplementary material for: Population structure and historical demography of South American sea lions provide insights into the catastrophic decline of a marine mammal population
Source: R Soc Open Sci. 2016 Jul 27;3(7):160291. doi: 10.1098/rsos.160291 (PMC4968474; doi:10.1098/rsos.160291)
Supplement: Electronic supplementary material, figure S1. Results of the Structure [38] analysis showing average log-likelihood value values based on five replicates for each value of K, the hypothesized number of clusters in the data. Electronic supplementary material, table S1. Numbers of sea lion tissue samp [file rsos160291supp1.docx]

**Electronic supplementary material, table S1.**

| Region | Colony | Literature reference | Number of samples collected | Number of samples genotyped at: | | |
| --- | --- | --- | --- | --- | --- | --- |
|  |  |  |  | Mitochondrial DNA | Microsatellites | Mitochondrial DNA and microsatellites |
| West Falkland | Circum Island | This study | 6 | 6 | 6 | 6 |
|  | North Fur Island |  | 1 | 1 | 1 | 1 |
|  | South Fur Island |  | 3 | 3 | 3 | 3 |
|  | Outer Island |  | 4 | 4 | 4 | 4 |
|  | Peat Island |  | 3 | 2 | 3 | 2 |
|  | Port Egmont Cays |  | 3 | 2 | 3 | 2 |
|  | Steeple Jason Islet |  | 3 | 3 | 3 | 3 |
|  | Stinker Island |  | 4 | 3 | 4 | 3 |
|  | Twins North |  | 2 | 2 | 2 | 2 |
|  | Twins South |  | 2 | 2 | 2 | 2 |
|  | West Double Creek |  | 2 | 2 | 2 | 2 |
|  | *Total* |  | 33 | 30 | 33 | 30 |
| East Falkland | Big Shag Island | This study | 126 | 123 | 123 | 120 |
|  | Blind Island |  | 13 | 10 | 13 | 10 |
|  | Cape Dolphin |  | 1 | 1 | 1 | 1 |
|  | Cattle Point Island |  | 3 | 3 | 3 | 3 |
|  | Green Island |  | 18 | 18 | 18 | 18 |
|  | Kelp Islands |  | 15 | 13 | 13 | 13 |
|  | Motley Island |  | 6 | 6 | 6 | 6 |
|  | Port Harrie |  | 15 | 14 | 14 | 14 |
|  | Sal Island |  | 1 | 1 | 0 | 0 |
|  | Sandy Tyssen |  | 7 | 7 | 7 | 7 |
|  | Turn Island |  | 32 | 32 | 32 | 32 |
|  | Tussock Point Island |  | 6 | 4 | 6 | 4 |
|  | West Tyssen |  | 1 | 1 | 1 | 1 |
|  | *Total* |  | 244 | 233 | 237 | 229 |
| Argentina | Punta Norte | [21] | – | 12 | – | – |
|  | Puerto Pirámide |  | – | 7 | – | – |
|  | Isla Arce |  | – | 10 | – | – |
|  | Isla Vernacci Oeste |  | – | 10 | – | – |
|  | Monte Loayza |  | – | 10 | – | – |
|  | *Total* |  | – | 49 | – | – |
| Brazil | Coastline along Rio Grande do Sul | [19] | – | 56 | – | – |
| Chile | Arica | Weinberger 2013, PhD dissertation (GenBank refs: JQ434428 to JQ434457) | – | 4 | – | – |
|  | Punta Pichalo |  | – | 6 | – | – |
|  | Punta Piojo |  | – | 2 | – | – |
|  | Punta Patache |  | – | 7 | – | – |
|  | Punta Negra |  | – | 7 | – | – |
|  | Pabellón de Pica |  | – | 4 | – | – |
|  | Punta Lobos |  | – | 5 | – | – |
|  | Punta Campamento |  | – | 3 | – | – |
|  | Bandurrias del Sur |  | – | 4 | – | – |
|  | Punta Bandurrias |  | – | 1 | – | – |
|  | Pan de Azúcar |  | – | 5 | – | – |
|  | Punta Obispo |  | – | 2 | – | – |
|  | Punta Cacho |  | – | 1 | – | – |
|  | Isla Chañaral |  | – | 2 | – | – |
|  | Coquimbo |  | – | 3 | – | – |
|  | Tunquén |  | – | 1 | – | – |
|  | Cobquecura |  | – | 1 | – | – |
|  | Metalqui |  | – | 8 | – | – |
|  | Rosa |  | – | 1 | – | – |
|  | Rómulo |  | – | 2 | – | – |
|  | Isla Marta |  | – | 2 | – | – |
|  | Punta Arenas |  | – | 1 | – | – |
|  | *Total* |  | – | 72 | – | – |
| Peru | Punta San Juan | [25] | – | 5 | – | – |
| Grand total |  |  | **277** | **445** | **270** | **259** |
